# Early-Onset Alzheimer Disease (EOAD) With Aphasia: A Case Report

**DOI:** 10.3389/fpsyt.2018.00469

**Published:** 2018-09-27

**Authors:** Marcus Kiiti Borges, Thais Nakayama Lopes, Marina Maria Biella, Alaíse Siqueira, Sivan Mauer, Ivan Aprahamian

**Affiliations:** ^1^Department of Geriatric Psychiatry, FEAES (Fundação Estatal de Atenção Especializada em Saúde), Curitiba, Brazil; ^2^Department of Geriatrics, Hospital das Clínicas da Universidade de São Paulo, ACID (Ambulatório de Alterações Comportamentais em Idosos), São Paulo, Brazil; ^3^Department of Psychiatry, FMUSP (Faculty of Medicine - University of São Paulo), São Paulo, Brazil; ^4^Department of Internal Medicine, FMJ (Faculty of Medicine of Jundiaí), São Paulo, Brazil

**Keywords:** Alzheimer's disease, early onset, dementia, aphasia, case report

## Abstract

**Background:** Alzheimer's disease (AD) is traditionally subdivided into early onset (EOAD) and late onset (LOAD). EOAD has an onset before age 65 years and accounts for 1–5% of all cases. Two main presentation types of AD are familial and sporadic.

**Case presentation:** The authors present the case of a 68-year-old retired white man, with a college level educational background. At 55 years of age, the patient presented cognitive decline with short-term memory impairment and slowed, hesitant speech. At 57 years, he was unable to remember the way to work, exhibiting spatial disorientation. PET-CT: revealed hypometabolism and atrophy in the left temporal lobe and posterior region of the parietal lobes.

**Disease course:** Evolving with difficulties in comprehension and sentence repetition over past 3 years and with global aphasia in past 6 months, beyond progressive memory impairment.

**Discussion:** Possibly due to the young age and atypical presentation, and the diagnosis of EOAD is often delayed. To the best of our knowledge, this case can be classified as a sporadic EOAD with aphasia. Clinical variant and neuroimaging findings were crucial to the diagnosis and treatment of this atypical presentation of AD.

## Introduction

A 68-year-old retired white man, married, and with college-level educational background presented at our inpatient service accompanied by his wife and daughter, reporting complaints of “aggressivity and intense agitation.”

He showed restlessness and disorganized behavior, and failed to recognize people (agnosia). He was unable to answer any questions because he could not understand what was being said (receptive aphasia) and did not adhere to clinical orientations or follow instructions given by the team, proving unable to perform basic motor actions (e.g., he refused to take the medications prescribed, “he grasped the pills and didn't take them”). Patients with receptive aphasia have impaired comprehension of their speech, thus they may have trouble answering basic orientation questions.

Results of complete blood count and coagulogram, biochemistry, renal, liver, and thyroid function tests, vitamin B12 levels, serology for syphilis and anti-HIV, qualitative urine test, urine, and blood cultures (2 samples), CSF profile and chest x-ray were all within normal limits. Computed tomography of the brain was performed on January 2018 and underlying neurologic conditions were ruled out.

## Background

### History

The patient was admitted to the emergency service on January 2018. The reason reported by the family members for the current admission was agitation “he can't keep still, walking back and forth the whole time” and aggressiveness “he tried to physically harm his wife and had to be kept away from mirrors and windows because he would try and shatter them.”

At 55 years of age, the patient first presented cognitive decline with predominantly short-term memory loss and slowed, hesitant speech. According to the reports of his wife and daughter he tended to forget where he had left his keys and had trouble remembering recent events such as appointments.

At 57 years, he was no longer able to remember the way to work, stopped driving, and exhibited spatial disorientation. He became apathetic and had difficulties taking decisions at work and was unable to manage his finances. At this time, due to social and occupational deficits, the family decided to seek a Neurologist for clinical assessment. In October 2009 (at 59 years), he underwent a Magnetic Resonance of brain which revealed a reduced volume of hippocampi, particularly the choroidal fissures [grade II/III on Scheltens et al. (1) scale]. After confirmation of a diagnosis of probable Alzheimer's disease (AD), he retired due to the disease.

Since 2013 (past 5 years after the diagnosis), he has been followed by a neurologist for treatment of cognitive symptoms with: donepezil 10 mg and memantine 20 mg. A follow-up Computed Tomography of the brain (August 2013) depicted bilateral (posterior) parietal atrophy, and most affected in the left temporal lobe. These regions were markedly atrophied.

**Figure 1:**
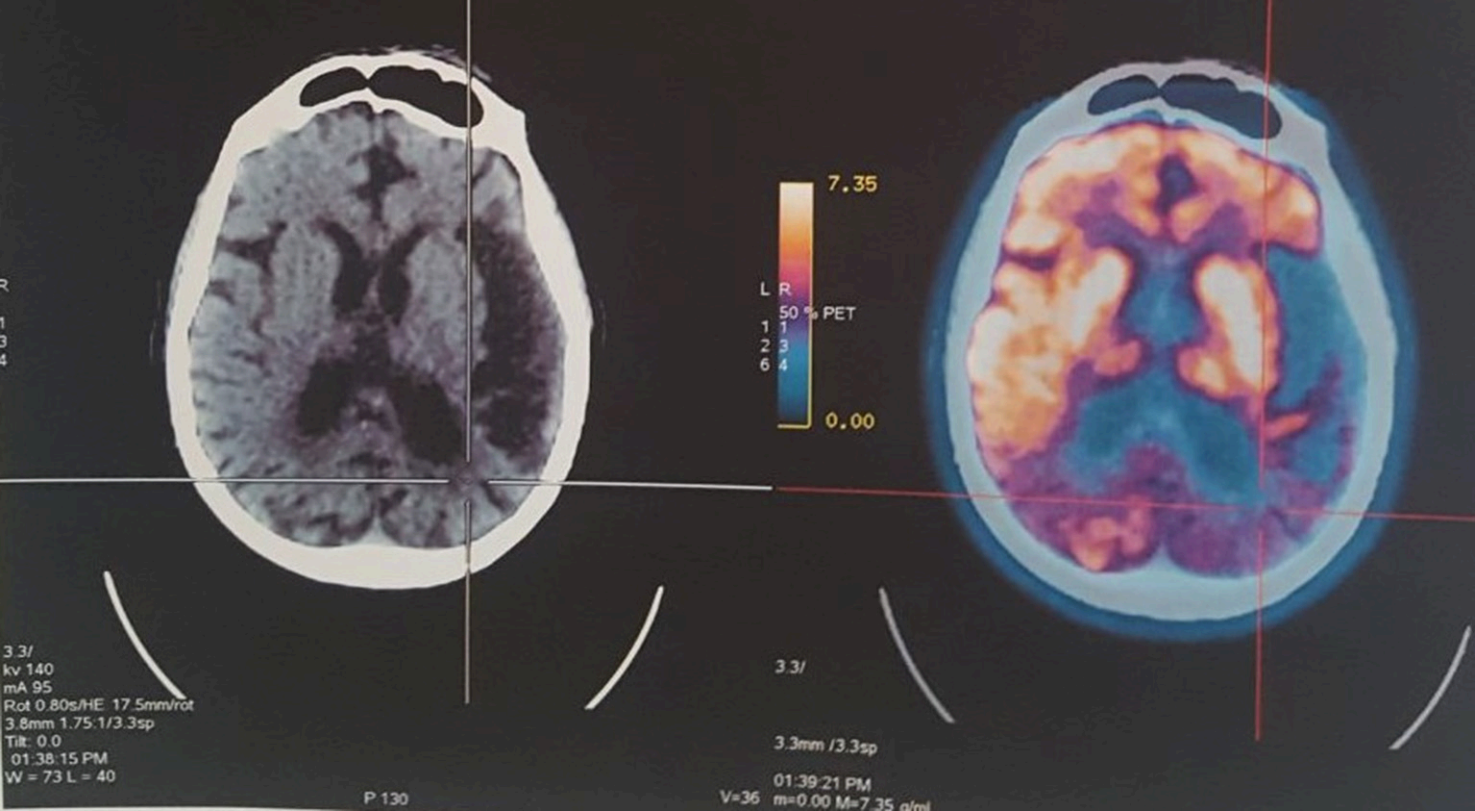
PET-CT revealed hypometabolism and atrophy in the left temporal lobe and posterior region of the parietal lobes.

A PET-CT scan (July 2015) showed signs of diffuse symmetrical volume reduction of the parenchyma in the cerebral hemispheres. Bilateral hypodensity of the periventricular white matter was evident. Hypometabolism was observed in the left temporal lobe and posterior region of the parietal lobes, diagnosis of probable EOAD (Figure 1).

The patient presented hypertension, dyslipidemia and smoking previously, and unremarkable neuropsychomotor development. Also, he had no history of hospital admissions or surgery. Family members reported no history of neurodegenerative or psychiatric disease.

The patient evolved with difficulties in comprehension and sentence repetition over past 3 years and predominantly language deficits (global aphasia) in past 6 months. The patient has cognitive progressive decline when comparing his evaluations between January 2015: Mini-Mental State Examination (MMSE) = 13; Verbal Fluency Test (VFT) = 7; Clinical Dementia Rating (CDR) = 1; Katz—Index of Independence in Activities of Daily Living (ADL) = 4; Pfeffer—Functional Activities Questionnaire (FAQ) = 25; and January 2018: MMSE = not assessed; VFT = 0 (aphasia); CDR = 2, besides behavioral and psychological symptoms of dementia (BPSD), including: agitation, emotional lability, shifting between increased irritability or aggressiveness and apathy. The patient exhibited lack of initiative such as reduced interest in previously enjoyed activities. Inability to plan was another serious symptom. Occasionally the patient showed emotional blunting (with low emotional response).

He was taking losartan 50 mg, rosuvastatin calcium 10 mg, vitamin D, memantine 20 mg, donepezil 10 mg, venlafaxine 150 mg, and quetiapine 50 mg.

During the current hospital stay, in January 2018, he had a major episode of psychomotor agitation with risk of aggression. The patient was medicated with haloperidol and midazolam and physical restraints were applied for the patient's safety. The patient was distraught but showed no change in level of conscientiousness, and *delirium* was rejected.

The daughter assisted by providing a calm care environment, playing the patient's favorite song. Other measures taken were: promoting the patient's mobility (“taking him for walks around the woods in the hospital grounds”); increasing the level of lighting and reducing noise level in the room; and introducing protocols for sleep and fall prevention. Because the patient was in use of memantine 20 mg and donepezil 10 mg, quetiapine dose was increased from 50 to 400 mg (gradual escalation from 50 mg/day over 4 days). After no response to quetiapine, we switched to olanzapine 10 to 20 mg as alternative on the management of BPSD.

His wife reported that the patient was calm and more cooperative after adjusting medication and introducing the nonpharmacologic measures. Sleep quality improved and the patient walked with assistance, and no further chemical control or physical restraints were required up until hospital discharge on February 2018. The patient was referred for out-patient unit follow-up at Geriatrics and Geriatric Psychiatry services.

This study was carried out in accordance with the recommendations of the Declaration of Helsinki. Written informed consent was obtained from the patient for the publication of this case report. His anonymity has been preserved.

## Review of similar cases

AD has two forms, with an estimated 5–10% of patients having the “pure familial” type, while the remaining 90–95% is generally referred to as having the “sporadic form” (2).

In the past 20 years, a number of researchers have identified three genes (PSEN2, PSEN1, and PPA) that are defective in chromosomes 1, 14 and 21, respectively (3–5). Individuals with Alzheimer's mutations in any of these three genes tend to develop symptoms before age 65, early-onset Alzheimer's disease (EOAD), while the majority of cases have late-onset Alzheimer's disease (LOAD), in which symptoms appear at age 65 years or older (6).

Case reports of EOAD are scarce in the literature. A search of the PubMed databases using the terms: (“Alzheimer's Disease” AND “early-onset”) AND (“familial” OR “genetic”) for the past five years, retrieved 34 published reports, only 15 of which were considered early-onset or familial AD cases. Of these reports, 10 cases were associated with genetic changes or mutations in PSEN1, 3 in PSEN2, only 1 in PPA and another in APOE. This explains the disease observed in family members over successive generations, directly associated with a pattern of autosomal dominant gene transmission (7). Even though these familial mutations have been well established and they support genetic risk factor for AD, together they only account for about 50% of all familial AD cases, suggesting that additional genetic remain to be identified (2).

However, when the familial form is a possible diagnosis, there is no defined pattern of genetic inheritance (8). There may be no family history in many early-onset pre-senile AD patients, as seen in the case reported. These cases can be described as occurring due to so-called “*de novo”* mutations (9).

Although mutations in the known genes are a rare cause of AD, they are important for pre-symptomatic diagnostics of patients of autosomal dominant AD families that segregate these mutations. In sporadic cases, the ε4 allele of the apolipoprotein E (*APOE*) was indentified as a major risk factor contributing to the pathogenesis of AD in about 20% of the cases (10).

Sporadic AD is characterized by an isolated case in the family or cases separated by more than three degrees of relationship. Sporadic AD represents approximately 75% of all cases. Typically, sporadic cases are LOAD, but approximately 40% of EOAD cases may be classified as sporadic possibly representing hidden familial or autosomal dominant disease (11). This case report is considered a sporadic EOAD, despite the absence of family history and the fact that genetic and biomarkers tests were not performed in the patient. Legal representative of the patient didn't agree to proceed with genetic testing even after providing informed consent.

## Discussion

### Diagnosis

We reported a case of probable EOAD at a moderate-to-severe stage, with symptoms onset at 55 years of age (pre-senile onset) and atypical presentation.

Behavior variant frontotemporal dementia (bvFTD) is characterized by prominent changes in personality, interpersonal relationships and conduct. Behavior changes are often the first symptoms in bvFTD, however they tend to occur later in EOAD. Besides, problems with spatial orientation, for example getting lost in familiar places, are more common in EOAD than in frontotemporal dementia (FTD).

Aphasia is categorized as expressive (Broca) or receptive (Wernicke's). Many patients have a component of both types of aphasia. Degenerative brain disorders cause mainly Wernicke's aphasia. Primary progressive aphasia (PPA) is the second major form of FTD that affects language skills, such as speaking, writing and comprehension. Individuals with logopenic variant of PPA, lose the ability to understand or formulate words in a spoken sentence. In the 45 to 65 age range, both bvFTD and PPA are nearly as common as EOAD (12).

Logopenia is described as “mixed aphasia” because language expression is not fluent and understanding is impaired. In this case we reported the occurrence of receptive (Wernicke's) aphasia with logopenic characteristics in the course of EOAD.

Imaging studies investigated the overlap of the logopenic PPA and EOAD, comparing the pattern of cortical atrophy between AD variants (13, 14). Consistent with structural neuroimaging findings, extensive atrophy of the temporal lobe, predominantly in the left posterior perisylvian region (including Wernicke's area), and the results of functional imaging with FDG PET confirm the pattern of left temporoparietal hypometabolism in patients with the logopenic variant (14).

Common atrophy across variants was found in temporoparietal regions that comprise the posterior default mode network (DMN) (15, 16). At EOAD, atrophy patterns have largely converged across AD variants (17, 18). This pattern of damage is similar to that observed in early clinical stages of non-amnestic AD (19). Possibly due to the young age and atypical presentation, diagnostic accuracy is particularly low in EOAD and the diagnosis is often delayed (20).

### Treatment

The differential diagnosis of the dementias (FTD vs. AD) is vital for correct drug treatment. Treatment of moderate-to-severe AD consists of medications that affect the activity of the neurotransmitters acetylcholine and glutamate. Preliminary evidence of the DOMINO-AD study shows that this combination can delay the need for nursing home replacement of patient (21).

In addition, Howard et al. (22) demonstrated the value of maintenance treatment using donepezil, where patients randomly assigned to continue donepezil, switch to memantine alone or use both drugs (continue donepezil and start on memantine) showed benefits in cognitive and functional domains after 12 months compared to the placebo group. However, combined treatment for at least 12 months appeared not to be cost-effective compared with continuation with donepezil treatment alone, particularly in patients with advanced AD (MMSE < 10) (23). Moreover, continuation with donepezil treatment appeared to be more effective than discontinuation (24).

Experts recommend quetiapine as the most appropriate agent for all BPSD (25), as seen in the case reported this atypical antipsychotic was the preferred first-line agent. Unfortunately, the patient failed to respond to quetiapine, and for this reason, the treatment was switched to olanzapine. However, the use of antipsychotics to treat the behavioral symptoms of dementia is associated with greater mortality (26).

## Concluding remarks

Biologically, important subgroups of AD patients may respond to different treatments. In this patient, pharmacologic and nonpharmacologic measures were effective in treatment of acute and treatment-resistant BPSD. Clinical variant and neuroimaging findings were crucial to the diagnosis and treatment of this atypical presentation of AD.

## Ethics statement

This case study was carried out in accordance with the recommendations of the Ethical Committee of SMS—Curitiba (CAAE number: 93786618.9.0000.0101). An informed consent form authorizing the use of his clinical data for research purposes was signed by a responsible proxy.

## Author contributions

MKB, MMB, and IA substantial contributions to the conception or design of the work. MKB, TNL, AS, and SM the acquisition, analysis or interpretation of data for the work. MKB and IA drafting the work or revising it critically for important intellectual content. MKB, TNL, MMB, AS, SM, and IA final approval of the version to be published.

### Conflict of interest statement

The authors declare that the research was conducted in the absence of any commercial or financial relationships that could be construed as a potential conflict of interest.
